# “It has not occurred to me to see a doctor for that kind of feeling”: a qualitative study of Filipina immigrants’ perceptions of help seeking for mental health problems

**DOI:** 10.1186/s12905-018-0561-9

**Published:** 2018-05-25

**Authors:** Melanie L. Straiton, Heloise Marie L. Ledesma, Tam T. Donnelly

**Affiliations:** 1 Department of Mental Health and Suicide, Norwegian Institute of Public Health, P.O. box 4404, 0403 Oslo, Norway; 2 Department of Child and Adolescent Psychiatry, Oslo University Hospital, P.O. Box 4956, Nydalen, 0424 Oslo, Norway; 3 Faculty of Nursing, Cumming School of Medicine, Community Health Sciences, University of Calgary, 2500 University Drive NW, Calgary, AB T2N 1N4 Canada

**Keywords:** Mental health, Immigrants, Women’s health, Access to care, Barriers, Qualitative research

## Abstract

**Background:**

Immigrant women face greater barriers to health care, especially mental health care, than non-immigrant women. However, immigrants are a heterogeneous group and bring with them a range of different personal, social, cultural and economic factors, which impact both mental health and access to care. In this study, we explored factors that influence Filipina immigrants’ perceptions of help seeking from a general practitioner for mental health problems in Norway.

**Method:**

Using data from semi-structured interviews, we applied a post-colonial feminist perspective to identify factors that affect perceptions of help seeking.

**Results:**

Findings indicated that a combination of the women’s beliefs and values, stigma, experiences with healthcare services in Norway and familiarity with mental health services influence perceptions of help seeking. Some factors represented structural barriers to healthcare seeking in general, while others related to mental healthcare seeking in particular. The significance of each factor varied depending on the women’s backgrounds.

**Conclusions:**

Socioeconomic status, educational background, familiarity with health services and experience of mental health can influence immigrant women’s perceptions of, and barriers for, help seeking for mental health problems. There are a number of barriers to address at a structural level to improve both the propensity to seek healthcare in general, as well as mental healthcare in particular. Efforts to increase awareness of primary mental healthcare services may also help change the perception that professional help is only appropriate for serious mental health disorders.

**Electronic supplementary material:**

The online version of this article (10.1186/s12905-018-0561-9) contains supplementary material, which is available to authorized users.

## Background

Immigration from low and middle income countries to high income countries is increasing around the world [1]. Migration helps the economy, fills labour shortages in both high and low skilled occupations and contributes to social and economic stability in the host country [2]. However, immigrants may be at greater risk of mental health problems than the general population [3] due to the migration and adjustment process. They also have lower utilisation rates of health services for mental health problems [4–6]. Although entitled to the same social benefits as the rest of the population in many countries, documented immigrants may still face barriers in using healthcare services [7], which can contribute to declining health. Identifying barriers can improve health care for immigrants throughout the world.

Almost half of immigrants around the world are women [8]. Women have different migratory experiences than men and face greater social disadvantage. They are at higher risk of exploitation and abuse, experience poorer socioeconomic conditions and more social isolation [9]. These factors can both increase the risk of mental health problems and affect access to care. It is therefore important to address gender-specific migratory experiences in order to better understand and facilitate help seeking for mental health problems among immigrant women. In this study, we explore the contextual factors that influence perceptions of professional help seeking for mental health problems among Filipinas living in Norway.

### Norwegian health care

The Norwegian healthcare service is a universal, publically-funded system based on a principle of equity; equal access for equal needs. Long-term residents (over 6 months) and registered asylum-seekers are covered in the healthcare insurance system. It is organised in two main sectors; primary, which includes long-term care services, general practitioners and emergency care, and secondary which includes hospital and specialised services [10]. Residents are assigned a general practitioner (GP) who acts as a gatekeeper to specialised services. Majority of patients with mental health problems are treated at the primary care level [11].

GPs are usually self-employed and paid through a combination of the state, the municipality and patient co-payments. With the exception of children under 16 and pregnant women, patients pay a consultation fee when visiting their GP and other specialists, such as psychologists. Costs in excess of 2185 Norwegian kroner (260 USD) per year (around 10 consultations) are covered under the insurance scheme [12]. Hospital services are free to the patient. There is also a private system in Norway, where specialists can be accessed directly, without a referral from a GP but patient consultation fees are around four times higher.

### Filipinas in Norway

In Norway, Filipinas are the biggest group of immigrant women coming from outside of the European Union [13]. Filipino migration to Norway is highly feminised; almost 80% of immigrants from the Philippines are women. As well as fulfilling the demand for skilled care work such as nursing and unskilled, low paid domestic work, many Filipinas also marry Norwegian men [14].

In Australia, almost a quarter of Filipina immigrants reported significant psychological distress [15]. The prevalence of mental health problems among Filipina immigrants in Norway is not known. However, they are far less likely to have had a primary care consultation for a mental health problem than Norwegian women [16]. While the healthy migrant hypothesis, that immigrants have better health than the host population, could potentially explain this difference in health care use, studies in Norway actually suggest that immigrants report poorer mental health than non-immigrants [17]. In addition, the study on health care use among Filipina immigrants found that among women who do consult with a doctor, Filipinas are less likely to purchase psychotropic medicine or have conversational therapy with the general practitioner than Norwegian women [16]. This may suggest different healthcare needs, differences in treatment preferences, alternative methods of coping or the existence of barriers that prevent access to healthcare services and appropriate treatment.

### Barriers to care

Studies of health service use among various immigrant groups show that immigrants experience problems in accessing healthcare due to structural barriers such as cost, time and travelling, lack of knowledge of services and lack of appropriate services [18–21]. Language incongruence between the healthcare provider and the patient, as well as differing expectations about communication styles may present particular challenges in accessing care and appropriate treatment for women [21–23]. Specifically in relation to mental health problems, low awareness and stigma are also commonly cited cultural barriers among various groups [22, 24–26].

Immigrants are a heterogeneous group and bring with them a range of different personal, social, cultural and economic factors which impact health service utilisation. Studies specifically addressing Filipino immigrant populations in the USA indicate that loss of face is negatively related to both attitudes to professional help seeking and actual help seeking [27–29]. Low level of English proficiency may be associated with lower likelihood of help seeking [29]. It is noteworthy that majority of research with Filipino immigrants is from the USA. Migration policies and the structure of the health service also have an impact on how immigrants access healthcare services [30]. Thus we cannot assume that barriers to care are the same for all Filipino immigrants in different contexts.

Further, these studies with Filipino populations are quantitative and so the barriers the researchers investigated were pre-determined rather than identified by participants themselves. A qualitative study with Filipinas in Australia found that women often saw their mental health problems as temporary and situationally dependent, and thus not requiring intervention from a professional [31]. The women valued being self-sufficient in coping with their difficulties. By allowing women the opportunity to describe their own experiences and the meanings they attribute to them, we can better capture the complexity and diversity of their perceptions of help seeking [24]. In this way, the women themselves can identify issues that healthcare providers and policy makers should be aware of, in order to improve access to care.

In a previous paper, we explored the contextual factors that influenced the mental health of Filipinas living in Norway and ways of coping [32]. Uncertainty about the future was a common source of stress for women who only had short-term residency. They were often under-employed in low-skilled work despite being highly educated. The multiple, transnational roles that the women occupied placed additional pressure on them; roles as workers, as breadwinners, as wives, daughters and mothers. Some were also mothers separated from their children, which affected their mental health. The study showed that immigration policy can contribute to and maintain power imbalances, resulting in the marginalisation of immigrant women [32].

Informal support sources such as family in the Philippines and social support networks in Norway were utilised to a great extent and many of the women reported gaining strength from their religious beliefs. Despite some reporting significant levels of psychological distress, none of the women in the study had sought professional help and only one woman indicated an intention of doing so. To improve access to mental healthcare for immigrant women, it is important to understand the factors that hinder or facilitate help seeking. Given that the GP is the gatekeeper to mental healthcare in Norway, we asked: What factors influence Filipina immigrants’ perceptions of help seeking from a GP for mental health problems? To do this, we explored Filipinas’ experiences with, and views of, primary healthcare in Norway, as well as their perceptions of mental health.

## Methods

### Sample

The sample consisted of 14 informants, aged 24–49 years, who had been living in Norway between one and 6 years. Seven were married at the time of interview, six had children and all but one were employed and/or studying. Half of the women were working, or studying to work, in the health care sector. Ten had college or university level education. Four women had initially moved to Norway through the au pair scheme (a cultural exchange with a host family in return for domestic work and childcare [33]), four as job-seekers, four as wives/partners and two for other reasons. All but two of the women were covered by the Norwegian Health Insurance system. During the interview, five of the informants spoke about having experienced depression or anxiety at some stage in life. One experienced this for the first time while living in Norway. Five also scored above 1.85 on the Hopkins Symptom Checklist Scale (HSCL-10), a reliable measure of psychological distress [34]. This suggests they were experiencing significant levels of distress around the time of the interview.

### Interview procedure

We used purposive and snowballing sampling techniques to recruit potential informants [35] via key contacts in the Filipino community; members of associations, churches or other networks. We contacted key persons via telephone, e-mail or in person. Key persons then informed women in their networks about the study. Inclusion criteria included being over the age of 18 and having lived in Norway one to 10 years. Written information about the goals of the study, the interview procedure, informants’ rights and ethical considerations was issued to the women. We arranged suitable time and place for the interview with those who wished to participate. Following the interview, we asked informants to tell their contacts about the study and those who wished to participate contacted the first author. We obtained consent for voluntary participation from each informant. Informants’ rights, anonymity and confidentiality were assured.

Informants completed a short questionnaire with background information prior to interview (see Additional files 1 and 2). We then used a semi-structured interview guide with open-ended questions on topics such as living in Norway, family background, emotional difficulties, perceptions of mental health and experiences in consulting with general practitioners in Norway (see Additional file 3). The first author, a native English speaker, fluent in Norwegian, interviewed all the informants; thirteen in English and one in Norwegian. The women had the opportunity to request a Filipino interpreter but none did. Interviews were audio-recorded and lasted on average 60 min (range 25–100 min). They were transcribed verbatim, yielding data saturation with 277 A4 pages.

### Analysis

Our analyses were informed by a post-colonial feminist perspective. Post-colonialism is the resistance against the after-effects of colonialism; the struggle against inscribing inferiority to once colonised people [36]. However, the theory is considered male-centric. Post-colonial *feminism* (PCF) recognises that colonised women have a further struggle; to resist against the double inferiority ascribed through both colonisation and patriarchy [37]. PCF emerged through the recognition that mainstream Eurocentric feminism is not relevant for majority of women throughout the world. There is however, no single definition of PCF [38].

Anderson and McCann argue that post-colonial feminism can “shed light on the complex issues at the intersection of gender, race, class relations and culture, and further our understanding of how material existence ... influences health and well-being for those who… have [experienced] ... diaspora, [and] displacement’” ([39], p.11). This form of post-colonial feminism can critically address the experience of marginalised populations; whether it is in the past, present, or future and has a wide range of applications. It often involves the discussion of various experiences such as migration, suppression, resistance, differences, gender, race, as well as social discrimination and minority issues experienced by women who are socially and economically disadvantaged in contemporary societies. While all postcolonial feminist theorists attempt to speak for the ‘other’, they differ depending on their social location (in both time and space) [38].

In health care, PCF offers a theoretical framework to explore issues such as equity in health and accessibility in health care services at the time when global migration and health care reform are happening in many Western countries [38]. This form of critical enquiry challenges the assumptions of dominant society and highlights power imbalances that can create or reinforce inequity in health or healthcare services [39]. This PCF perspective is heedful of differences based on gender, ethnicity and socioeconomic position and how these are influenced by social, cultural, political, historical and economic factors that shape the lives of marginalised women [40]. In the present research, we recognise Filipina immigrant women’s marginalised voices as legitimate, as a direction for health care actions that are responsive to their specific social locations within Norwegian society; their positions as women, as immigrants, as Filipinos and (in some cases) of low socioeconomic status or the wives of Norwegian men. This perspective provides the analytic lens that enables us to critically examine these women’s narratives and identify their situations, gendered social relationships, and structural barriers that may contribute to, and reinforce, the problems of, immigrant women’s utilisation of public health care services in Norway.

With a post-colonial feminist perspective as a lens, the first author read and reread the transcripts to allow familiarity with the data. She noted initial impressions about each case and then coded the relevant data (referring to health, mental health and health services), case by case. By comparing and contrasting the codes across the cases, the first author developed them into higher order categories and presented them to the other authors. Through discussions and referring continuously back to the data, we then added substance to these categories by triangulating them with existing literature, until they evolved into larger themes. During all stages of analysis, we were mindful of the larger socio-cultural context, as well as what we knew about each of the women’s lives. NVivo was used to assist in the coding and categorising of the transcripts.

## Results

Our findings are organised into three main themes: *Beliefs and values, Familiarity with (mental) healthcare,* and *Healthcare experiences in the new country*. These themes cut across both mental health help seeking and general help seeking, as we saw that the factors affecting healthcare seeking in general were even more prominent for mental healthcare seeking in particular. Several of the sub-categories are two-sided; being both facilitators and barriers to help seeking, depending somewhat on the women’s personal circumstances and position in society and they include factors at the personal, sociocultural and structural levels.

### Beliefs and values

#### Beliefs about the causes of mental health problems

Our discussions focused mostly on mild to moderate mental health problems, including stress and emotional difficulties, depression and anxiety, although some of the women also talked about more severe psychiatric disorders. These were understood as having biological causes, being more enduring and present from early childhood. In some cases, people with severe disorders were understood to be a danger to themselves or others and professional help was seen as relevant.

A couple of informants mentioned biological causes, such as an imbalance in hormones. Most participants however, drew on psychological and social explanations for common mental health problems. Depression for instance, was perceived as a result of a difficult social situation, a traumatic event such as rape, war conflict or loss of a loved one, or a build-up of stress together with rumination. For many, depression was often attributed to, or equated with, loneliness or boredom, sometimes in addition to environmental causes such as the cold and dark climate of a Norwegian winter. Thus, several informants viewed depression as a temporary state that could be overcome in a short time. A number of informants felt depression was more common in Norway than in the Philippines due to lower levels of social support and greater social isolation:

*I think it’s more here, the depression. Because…mostly I notice in Norway you have a big house but you’re living alone. In the Philippines, I think not much because you have a small house but it’s ten of you and you have somebody to talk [to].*

Several women explained that awareness of depression in the Philippines was low and it was often not taken seriously: *‘A lot of people misunderstand what [depression] is. And if you say you have it they just think that: ‘nah, you’re just down’* and as a result, professional help was deemed unnecessary. Yet, at the same time, the women recognised that unaddressed mental health problems could have serious consequences, such as suicide. It was at this stage the women believed professional help would be beneficial.

A few women also mentioned culturally specific beliefs about the causes of mental health problems, such as hot and cold imbalances and evil spirits. However, they considered this to be a belief held in rural areas in the Philippines and tended not to endorse these explanations themselves. A number of the women indicated holistic views of health, where there is less distinction between body and mind, compared with biomedicine. They highlighted the important role of social support in maintaining mental, physical and spiritual health.

#### Filipino resilience

Openness to professional help seeking did not seem to relate to the degree to which the women experienced emotional difficulties. As described in a previous paper, most of the informants who had experienced a mental health problem utilised their strong support networks and gained strength from their religious beliefs to help them through their difficulties [32]. Having overcome previous depressive feelings without professional help, the women believed they could do so again.

*First I would deal with it the ways I did [before]. Because I think in a way, I will not go through that phase. Because before going to that phase I would have probably thought … What’s wrong with me? Why am I like this… So probably [because of] my experience already…I know how to deal with it*.

The informants positioned themselves and many of their fellow Filipinas as strong and adaptable: *‘Filipinos are very tough. I mean, even though we are sad, we still have a smile… we can still keep happy’.* They attributed this resilience to having developed a high threshold for problems due to having experienced difficulties throughout their lives. They compared this to Norwegians, who they saw as fortunately unburdened by problems such as poverty and natural disasters, and therefore less equipped to cope when encountering difficulties. The women valued self-care and self-reliance and believed they were able to cope without professional assistance:

*I am from Asia so I don’t (seek help) because I think that we are…I see a lot of problems. So therefore it is easier for us to get over them and move on… We are not just ‘oh no, I can’t do this’. Maybe we get a little depressed for example, for a month, but after we get on with things. We don’t need to go to the doctor and just talk about it, no... Because I heard here that if someone is depressed, they go to the doctor right? Talk about what the problem is … But for me, I am from the Philippines, no, I don’t do that. I need to try myself. I can do it myself.*

They also felt that Filipino family ties and social networks were stronger than Norwegian ones. Thus Filipinos were both less likely to experience mental health problems and better equipped to cope without professional help:

*We have our family as our back up system and we are close-knitted. One’s problem – my problem is everybody’s problem… in a way they help each other and if we have a very good backup system, you’re being uplifted …. We talk it out, we have family.*

In the case of events such as death of a loved one, the women implied that in the Philippines, people would move on through time with support from family and friends and comfort and strength from their faith. As such, the women normalised their experiences and did not see a role for professional intervention, even when suffering from depression:

*I guess at that time I didn’t think of myself as needing to go to a doctor. It’s more on… it’s me who has to do something about it. Because everybody goes through that, losing a loved one. Then they have overcome it, so why not me?*

#### Stigma

Most of the informants discussed stigma in relation to mental health problems, which was considered stronger in the Philippines. The women mentioned different aspects of stigma, each of which can help explain their reluctance to seek help. Firstly, mental health problems were often associated with madness, which made people fear the affected person: *It’s like: “Oh, what happened? Are you crazy? … Or are you hallucinating? Are you having delusions? Are you sick? Oh … you are dangerous”.* Secondly, seeking help for mental health problems meant the admission of not coping. This was doubly stigmatised because an individual was not only be labelled as crazy, but also unable to handle problems, undermining their sense of strength and resilience:

*It’s also wise to go to the doctor and have yourself checked. But not so many people want to do that. No. Also it’s kind of like a weakness, if you say that you have it, it’s kind of like saying: urgh, I can’t deal with this, so I’m weak.*

Thirdly, stigma related to mental illness extends beyond the individual and threatens to affect the family’s reputation: *‘Actually… our reputation is the thing. Especially, if you’re in high status … and people will start talking about you, so that wouldn’t be...a good thing*’.

As a result of multifaceted stigma, a few women explained that some Filipinos may be inclined to keep their problems hidden. The belief that mental illness reflects poorly on family lineage is not uncommon in Asian cultures. Preserving the public appearance of oneself and family, or ‘Saving face’ can lead to the concealment of mental health problems [41].

Many of the women distanced themselves from these stigmatising opinions. This particularly for women who had previously experienced a mental health problem, had an educational background in nursing or healthcare or otherwise increased awareness of mental health problems. Some informants had witnessed others seeking care from a GP for depression and this familiarity helped to reduce the stigma associated with help seeking: *‘so I said that...if in the Philippines, no [to help seeking]… but because here if you go to the doctors, since I am with my host mom before, she’s so depressed, yeah. I think, I mean it’s fine*’.

### Familiarity with (mental) health services

Public health approaches to mental healthcare are uncommon in low-income countries [42]. The informants indicated that mental health services in the Philippines are centralised, predominantly inpatient care and expensive. Many of the women were therefore unfamiliar with the idea of primary mental healthcare, which may explain why seeking help from the GP for less severe mental health problems was not a consideration for the informants: *‘It has not occurred to me, to see a doctor for that kind of feeling’.* Instead the informants mostly associated mental health care as something only useful in severe cases where, for example, the individual was considered a danger to themselves or others: *‘if that person [is] really hurting other people… you’d maybe call a professional or send them to mental hospital.... If it’s really bad’.*

Additionally, some informants were unsure how their GP could help them, though most generally suggested that the GP might give them advice, medication, therapy, or refer them to someone for further help. Although a couple of the women considered medication acceptable *‘if it’s really necessary. If there’s some, nervous breakdown,’* majority who mentioned medication did not like the idea of taking it themselves. They worried about addiction and side effects. As such, this may also increase reluctance to visit a GP:

*I suggest that before going [to see] a doctor for…depression… maybe you just have to… see for yourself if you can make it without… taking some pills, because it is really hard to take pills, because in the future…without pills, you cannot control yourself.… Things are much better if you [try] the natural way …*

Furthermore, several informants explained that it is common for many Filipinos to use alternative care in the Philippines (based on folk beliefs, herbal medicines and traditional healers) [43] instead of Western medicine for general health problems. Additionally, because the cost of healthcare in the Philippines can be high [43] several women indicated that they refrained from or delayed seeking general healthcare when they lived in the Philippines:

*In the Philippines, I haven’t really experienced consulting the doctor except… when I was younger when I got admitted in a hospital. So everything [is] serious when we see the doctor….It takes really long time to decide for us that this condition is serious. We don’t want to use our money right away.*

Thus, lack of familiarity with doctors in general may also explain why the women tend not to see GPs in Norway as a source of help, unless all other avenues are exhausted.

### Healthcare in the new country

The amount and type of experience that our informants had with the healthcare system in Norway varied. Since none of the women had sought help for mental health problems, we focused on exploring women’s perception and experience of consulting with the GP in general. Factors that appeared to affect perceptions of help seeking can be categorised into *access*, *language* and *GP-patient relationship*.

### Access

#### Inaccessibility of healthcare information

Learning to navigate the health service in a new country can be challenging for immigrants. The women came from a system with no GP or appointment system and were used to accessing immediate help directly from specialists. Thus, the gatekeeping role was new. When an immigrant is assigned a GP, they are informed by a letter written in Norwegian and so they consult others, such as spouses or in the case of au-pairs, their ‘host family’ (essentially their employer) for more information. This places Filipinas in a position of dependence on their Norwegian spouses or employers, reducing their autonomy. However, not all the married women got the help they needed: *‘he doesn’t explain to me everything about doctors, because he works every day… He‘s not home every day. He is away a lot. So I don’t know a lot about here…’*. Further, this informant, who had heightened child-care responsibilities in her husband’s absence, was also financially dependent on her husband. Dependence on a partner may leave immigrant women with less control over their own health, leading to delays in healthcare seeking.

#### Cost of services

The GP consultation fee was a barrier for a few of the informants. This related somewhat to their financial situation; they faced the double burden of low-socioeconomic status combined with financial responsibilities for their families back in the Philippines [32]. These women were forced to prioritise between visiting the GP and sending money to support their families in the Philippines. The strong sense of family responsibility meant that some women neglected their own health. One informant, who was studying full-time, working part-time and supporting a family at home, felt she could not spare time or money to attend her GP. Sadly, she was the only informant who also wanted to talk to a professional about her emotional problems but she worried about the cost:

*[it’s] expensive here, that’s the reason. I mean you cannot do anything instantly, because you need money. Yeah, especially me, I am a student. Instead of going there, [I think] okay I will just keep [the money], for this, for that.*

Furthermore, immigrants who have not yet been granted residence are not covered by the Norwegian insurance scheme. Although entitled to emergency care, one woman was unsure how to access primary healthcare before being assigned a GP. As a result, she had delayed visiting a doctor for a prescription of a regular medicine she needed. This highlights a major barrier to help seeking for immigrants whose residency is pending. In cases where women are applying for family reunification visas, as some informants had, they may be financially reliant on their husband or other family member for deciding if, and when, to access care. This again leaves them with less autonomy over their own health.

#### Language

The informants had varying levels of Norwegian language skills. Almost all were proficient in English. Since English is a second language in Norway and spoken with high proficiency [44], it is commonly employed when one party does not speak Norwegian. As a result, informant’s consultations with a GP usually consisted of a mix of English and Norwegian. The women indicated that this could sometimes lead to confusion, though they were able to resolve misunderstandings easily*: ‘I can manage to explain… and then we come up to this: “aah, you mean like that!” Yes!’* Some acknowledged however, that if they had had more serious health concerns, they may have felt differently. Only one woman, who actually had a Filipina GP, mentioned how important speaking the same language was for their communication:

*I could express myself in my language, so she knows exactly what I feel. Because if I say it in Norwegian, it would… not be accurate. I could miss the details…that I would actually like to say… It is… very beneficial.*

Although the women in this study did not feel language was a major problem, communicating in a second language is arguably not the same has having the ability to express thought processes in ones’ native language [45]. This may be of particular importance in instances that relate to emotional or psychological health.

Although immigrants in Norway can request an interpreter at a consultation, not all of the women were aware of this, and none had used the service. This supports the earlier suggestion that health information in Norway is not always accessible to immigrants.

#### Patient-GP relationship

In Norway, the emphasis during consultations is on patient involvement and shared decision-making [46]. In this model, a patient should be informed about alternative treatment options by, discuss their concerns with, and indicate their preferences to, the GP. A number of women were comfortable in this role and engaged in shared decision-making:

*She [the GP] is actually very willing to listen... I get my medications … we would also discuss it, so I would ask her, because I don’t like [to use] that… I took supplements like this, and I cannot drink this or that, so maybe we can find another one. She is really willing to listen.*

These women tended to have a healthcare background, which they felt helped them understand health information and talk with the GP as an equal partner. They took an active role in learning about their health and often consulted online sources. However, not all the women were comfortable with this role and expected a more authoritative GP figure who could tell them what was wrong, explain why and how to prevent further problems. When the GP did not have an answer, the women doubted the GP’s competence and trustworthiness:

*It is important to us that the doctor is ‘okay this is the reason why you are feeling this, why you are feeling that. Because they are the doctor, I mean we trust them…the doctor should know what the patient needs.*

Rapport with GP was another aspect the informants talked about. Some commented that their GP was approachable, helpful, non-judgemental and that they could easily open up to them but this was not the experience for all informants:

*Sometimes I feel like… my doctor’s in hurry. It’s like: “what your problem” and then ‘oh, you need— ‘there’s a note there already for medicine….Actually I think he is good, but I think he has lot of patients…. I can ask a little bit, but I can see in his body language that he is in hurry. So that’s the one negative thing.*

According to research with Filipino American’s [47], Filipinos may hide emotions and needs from people they consider to be ‘ibang tao’ (not one of us) and instead respond politely at a superficial level. In contrast, they are more likely to trust someone who they view as ‘hindi ibang tao’ (one of us). As such, Filipinos may find it easier to discuss emotional problems or distress with a GP who, although not from their own culture, is approachable, respectful and willing to accommodate. They are more likely to consider those GPs as ‘hindi ibang tao’. In light of this, short appointment times and doctors who have a direct style of communication can pose a barrier to seeking help for mental health problems:

*Well probably because when you think of your doctor, you think that you can tell her you know, your health problems and stuff, I guess the relationship is quite different …in our country. When you feel at ease with your doctor, that you can talk to them. That it’s not that they’re in a hurry, not listening to you.*

## Discussion

In this study, we aimed to identify factors that influence Filipinas’ perceptions of help seeking from a GP for mental health problems. In doing so, we also identified a factors relating to professional help seeking in general. The findings indicated that a combination of the women’s beliefs and values, associated stigma, experiences with the healthcare service in Norway and unfamiliarity with (mental) health services may all influence whether or not a GP is considered as an appropriate source of help. Through a post-colonial feminist lens, we saw that the significance of each barrier varied somewhat depending on the women’s own backgrounds, education, socioeconomic position and healthcare experiences. It should be noted that the women were active in seeking alternative coping strategies for dealing with mental health problems as described in a previous study [32]. At the same time, their strongest support - the family network - was weakened while living in Norway, heightening their vulnerability to mental health problems. Thus, the importance of professional help seeking becomes even more salient in this context.

There are a few methodological considerations to highlight in relation to the findings. Barriers can be perceived differently depending on one’s own need. Five of our informants were currently experiencing mental distress but only one informant in our sample had the desire to seek care. We may have identified other structural barriers had more women felt the need for care. Further, majority of our informants were well-educated and half of them worked, or were studying to work, in healthcare. These women were therefore more familiar with the health care system in Norway and less likely to experience problems regarding access to care than other Filipinas. We attempted to circumvent this in the findings by highlighting instances that applied mostly to those working in/studying healthcare. Additionally, through a post-colonial feminist lens, the inclusion of women with both higher and lower educational backgrounds meant that we were able to draw attention to how perceived barriers and facilitators varied according to the women’s resources and positions in society.

The first author conducted all the interviews and took a lead role in the analysis. As a British woman living in Norway, she shares some experiences with the informants in terms of being an immigrant and being a woman. This may have helped to gain the trust of the informants and make some more comfortable during the interview. Indeed, several of the women indicated some degree of identification: ‘*We are both foreigners, right?*’or ‘*you know, us women’*. However, not being a visible minority, together with coming from a different cultural background, different educational background (psychology), different health system and having the role as a researcher will have maintained a power differential during the interviews. At the same time, being an outsider to some extent can be advantageous; informants may elaborate more on topics they think the researcher does not know much about, yielding richer data for analysis [48].

Another concern is that the voice of the ‘other’ can be misrepresented due to the researchers own position during analysis and interpretation of the findings [39]. However, the other authors, both with Asian immigrant backgrounds and experience in health care settings were involved in the interpretation. This helped us to explore different perspectives and attempt to challenge the preconceptions of the dominant ‘Eurocentrism’ discourse. To enhance transparency, we provided a clear description of the informants, stages of data collection and analysis. We were rigorous by recording and transcribing the interviews and reporting the informants’ contrasting perspectives when findings did not apply to everyone.

We can draw a number of implications from this study that relate to both healthcare seeking in general and mental healthcare seeking specifically. First, a central aim of the Norwegian healthcare policy is to offer equal access for equal needs, regardless of a person’s background or position in society [49]. Yet, through using a post-colonial feminist lens, we were able to identify some power imbalances affecting equity to health care. Filipina women experience a number of structural barriers when accessing care as a result of their position as immigrants in society. At first, the women lack familiarity with the healthcare system and are often reliant on a partner or host family for information. These circumstances force the women into a subordinate position rendering them with less control over their own health. It is imperative that access to healthcare information is improved in order to empower immigrant women. This is important in terms of how the health system works, the right to healthcare and to interpreters.

Some of the informants in our study reported delaying seeking care due to consultation fees, particularly among those with low socioeconomic status. This supports research findings from other countries with so-called universal health systems [7, 18, 50]. While the cost of consultation fees for the patient are not considered high in Norway (relative to the cost of living/average salaries), the relative cost can be substantial for immigrants who work in low-paid sectors and who provide financial support to their families in their home country. Delayed help seeking can mean that health problems, which could be easily resolved at an early stage, may require more complex care when help is obtained at a later stage. This may be the case for mental health problems in particular, where women’s beliefs, values and familiarity with doctors can also inhibit help seeking. Consultation fees should be addressed at the structural level.

Language proficiency is an important factor in relation to healthcare experiences [23, 24]. Most of the women in this study were proficient in English and felt that they could communicate effectively with their GP, but some did suggest that language would be an issue if their health concerns were more complex. Given the complexity of mental health problems and the reliance on language for both diagnosing and managing them, subtleties in language can affect the transfer of information, explanation of diagnosis and discussion of treatment options. Communication difficulties, together with feelings of being rushed by their doctor as the women also described, can hinder women discussing their concerns about treatment options. It is essential that the GP ensures the patient fully understands the information shared in a consultation and that immigrants are made aware of their rights to request an interpreter during health consultations.

Further, some of the women who were less comfortable with shared decision-making may lack power in consultations. Studies on Filipino Americans suggest many Filipinos experience feelings of inferiority to those in positions of power due to their colonial history [51]. Thus, the power differential between a Norwegian doctor and an immigrant Filipina may make it difficult for some Filipinas to question their GP when unsatisfied with the amount, or quality, of information given. Without concrete answers or clear instructions, the patient may perceive the GP as incompetent and be less likely to consider them as a resource for help, especially when under time constraints. Again, this is especially the case for mental health problems, where Filipinos are more likely to open up to a person they feel comfortable with and can trust [47]. It may be that some immigrants require longer consultations in order to discuss their health concerns in a constructive and comfortable way. The GP should try to elicit the patient’s view about the cause of the problem, their previous coping strategies and their treatment expectations in order to make a culturally competent assessment and successful treatment of the problem [41].

Another noteworthy finding is that some Filipinas are also familiar with traditional medicine and many prefer herbal treatments over biomedicine. Several of the informants made suggestions of a holistic understanding of health; integration of the body, mind and spirit. Such cultural belief systems often include a range of preventative measures which may discourage reliance on biomedical systems [52]. It is important for health professionals to be aware of, and to encompass holistic understandings when discussing treatment options. Additionally, turning to religious leaders or gaining strength from one’s faith were important coping strategies for the women [32]. Health service cooperation with traditional healers and religious leaders may be useful for health prevention, promoting good mental health among immigrant groups and encouraging professional help seeking where appropriate [45].

Stigma is a frequently cited barrier to professional help among many immigrant groups [24–26]. Since the women in our study valued self-care and considered their fellow Filipinos resilient, they also indicated a personal fear of being weak. However, several women knew others who had sought professional help for depression, which appeared to make help seeking more acceptable to them. Researchers suggest that contact or familiarity with people who use mental health services is an important driving force in reducing stigma [53, 54]. Some researchers also link stigma to understandings of mental health and suggest that stigma may be reduced through education [54]. Educational campaigns should be delivered by and directed towards Filipinos, and include individuals with mental health difficulties in the program delivery. Such a campaign should also aim to increase the awareness of the availability of low-threshold services in Norway, in order to change the perception that intervention is only appropriate for serious mental health disorders.

## Conclusions

In this study, using a post-colonial feminist lens, we attempted to show how being a woman, an immigrant from the Philippines and (in many cases) of lower socioeconomic status can shape health experiences and access to care. We highlighted contextual factors that influence Filipina immigrants’ attitudes to professional help seeking for mental health problems and identified a number of barriers to help seeking at social, cultural and structural level. Structural level barriers relate to healthcare seeking in general. While identified barriers are in line with previous research with other groups of immigrant women, our detailed analysis adds insight into the different ways these factors can influence help seeking. A woman’s socioeconomic status, educational background, familiarity with health services, familiarity with language and experience of mental health are all important considerations when attempting to understand perceptions of, and barriers for, help seeking for mental health problems. There are a number of barriers that need to be addressed at a structural level in order to improve both the propensity of healthcare seeking in general, as well as mental healthcare seeking in particular.

## Additional files

Additional file 1: Interview guide (English): List of questions and probes used during interviews. (DOCX 17 kb)

Additional file 2: Questionnaire (English): Questionnaire for collecting background information. (DOCX 20 kb)

Additional file 3: Questionnaire (Norwegian): Questionnaire for collecting background information. (DOCX 20 kb)

## Abbreviations

GP: General practitioner
PCF: Post-colonial feminism

## References

[1] OECD. International migration outlook 2017 [internet]. Paris: OECD Publishing; 2017 [cited 2017 May 15]. Available from: http://www.oecd.org/migration/international-migration-outlook-1999124x.htm.

[2] OECD. Is migration good for the economy? [internet]: OECD, International Migration Division; 2014. (Migration Policy Debates). Available from: http://www.oecd.org/migration/OECD%20Migration%20Policy%20Debates%20Numero%202.pdf

[3] Lindert J, von Ehrenstein OS, Priebe S, Mielck A, Brähler E (2009). Depression and anxiety in labor migrants and refugees–a systematic review and meta-analysis. Soc Sci Med.

[4] Abe-Kim J, Takeuchi DT, Hong S, Zane N, Sue S, Spencer MS (2007). Use of mental health-related services among immigrant and US-born Asian Americans: results from the National Latino and Asian American study. Am J Public Health.

[5] Kirmayer LJ, Weinfeld M, Burgos G, du Fort GG (2007). Use of health care services for psychological distress by immigrants in an urban multicultural milieu. Can J Psychiatr.

[6] Straiton ML, Reneflot A, Diaz E (2014). Immigrants’ use of primary health care services for mental health problems. BMC Health Serv Res.

[7] Asanin J, Wilson K (2008). “I spent nine years looking for a doctor”: exploring access to health care among immigrants in Mississauga, Ontario, Canada. Soc Sci Med.

[8] OECD, UNDESA. World migration in figures [internet]: OECD-UNDESA; 2013. [cited 2015 Oct 18]. Available from: https://www.oecd.org/els/mig/World-Migration-in-Figures.pdf

[9] Llácer A, Zunzunegui MV, del Amo J, Mazarrasa L, Bolůmar F (2007). The contribution of a gender perspective to the understanding of migrants’ health. J Epidemiol Community Health.

[10] Romøren TI, Torjesen DO, Landmark BF. Promoting coordination in Norwegian health care. Int J Integr Care. 2011; [cited 2016 May 19];11(10). Available from: https://brage.bibsys.no/xmlui/handle/11250/142460 10.5334/ijic.581 PMC3226017 22128282

[11] Mykletun A, Knudsen AK, Tangen T, Øverland S (2010). General practitioners’ opinions on how to improve treatment of mental disorders in primary health care. Interviews with one hundred Norwegian general practitioners. BMC Health Serv Res.

[12] Health Norway. User fees for health services [Norwegian] [Internet]. 2017 [cited 2018 Mar 19]. Available from: https://helsenorge.no/egenandeler-for-helsetjenester.

[13] Statistics Norway. Immigrants and Norwegian-born to immigrant parents, by sex and country background 1970 - 2018 [Internet]. 2018 [cited 2018 May 02]. Available from: https://www.ssb.no/en/statbank/table/07108?rxid=e55fcbe0-a146-4b4f-a3ae-b4906d339062.

[14] Sandnes T, Østby L. Family migration and marriage patterns 1990–2013 [Norwegian] [Internet]. Oslo: Statistics Norway; 2015 [cited 2015 Oct 12]. Report No.: 2015/23. Available from: http://www.ssb.no/befolkning/artikler-og-publikasjoner/_attachment/228459?_ts=14d70308ab8.

[15] Thompson S, Hartel G, Manderson L, Woelz-Stirling N, Kelaher M (2002). The mental health status of Filipinas in Queensland. Aust N Z J Psychiatry..

[16] Straiton ML, Powell K, Reneflot A, Diaz E (2016). Managing mental health problems among immigrant women attending primary health care services. Health Care Women Int..

[17] Blom S, Vrålstad S, Stabell Wiggen K (2017). Health. Living conditions among immigrants in Norway 2016 [Norwegian] [Internet].

[18] Dias S, Gama A, Rocha C (2010). Immigrant women’s perceptions and experiences of health care services: insights from a focus group study. J Public Health.

[19] Donnelly TT, McKellin W, Hislop G, Long B (2009). Socioeconomic influences on Vietnamese-Canadian Women’s breast and cervical Cancer prevention practices: a social Determinant’s perspective. Soc Work Public Health.

[20] Scheppers E, Van Dongen E, Dekker J, Geertzen J, Dekker J (2006). Potential barriers to the use of health services among ethnic minorities: a review. Fam Pract.

[21] Thomson MS, Chaze F, George U, Guruge S (2015). Improving immigrant populations’ access to mental health Services in Canada: a review of barriers and recommendations. J Immigr Minor Health.

[22] O’Mahony JM, Donnelly TT (2007). “The influence of culture on immigrant Women’s mental health care experiences from the perspectives of health care providers”: erratum. Issues Ment Health Nurs.

[23] Suurmond J, Seeleman C (2006). Shared decision-making in an intercultural context: barriers in the interaction between physicians and immigrant patients. Patient Educ Couns.

[24] Donnelly TT, Hwang JJ, Este D, Ewashen C, Adair C, Clinton M (2011). If I was going to kill myself, I wouldn’t be calling you. I am asking for help: challenges influencing immigrant and refugee women’s mental health. Issues Ment Health Nurs..

[25] Sisley EJ, Hutton JM, Louise Goodbody C, Brown JS (2011). An interpretative phenomenological analysis of African Caribbean women’s experiences and management of emotional distress. Health Soc Care Community.

[26] Wu MC, Kviz FJ, Miller AM. Identifying individual and contextual barriers to seeking mental health services among Korean American immigrant women. Issues Ment Health Nurs. 2009;30(2):78–85. 10.1080/01612840802595204 19212865

[27] Baello J, Mori L (2007). Asian values adherence and psychological help-seeking attitudes of Filipino Americans. J Multicult Gend Minor Stud.

[28] David EJR (2010). Cultural mistrust and mental health help-seeking attitudes among Filipino Americans. Asian Am J Psychol.

[29] Gong F, Gage S-JL, Tacata LAJ (2003). Helpseeking behavior among Filipino Americans: a cultural analysis of face and language. J Community Psychol.

[30] Lindert J, Schouler-Ocak M, Heinz A, Priebe S (2008). Mental health, health care utilisation of migrants in Europe. Eur Psychiatry..

[31] Thompson S, Manderson L, Woelz-Stirling N, Cahill A, Kelaher M (2002). The social and cultural context of the mental health of Filipinas in Queensland. Aust N Z J Psychiatry.

[32] Straiton M, Ledesma HM, Donnelly TT (2017). A qualitative study of Filipina immigrants’ stress, distress and coping: the impact of their multiple, transnational roles as women. BMC Womens Health.

[33] Bikova M, Kontos M, Bonifacio G (2015). Au pair arrangement in Norway and transnational organization of care. Migrant domestic workers and family life: international perspectives.

[34] Strand BH, Dalgard OS, Tambs K, Rognerud M (2003). Measuring the mental health status of the Norwegian population: a comparison of the instruments SCL-25, SCL-10, SCL-5 and MHI-5 (SF-36). Nord J Psychiatry.

[35] Speziale HJS, Carpenter DR (2010). Qualitative research in nursing: advancing the humanistic imperative. %th ed.

[36] Quayson A. Postcolonialism: theory, practice or process. Cambridge: Polity Press; 2000.

[37] Tyagi R. Understanding postcolonial feminism in relation with postcolonial and feminist theories. Int J Lang Linguist. 2014;1(2) Available from: http://ijllnet.com/journals/Vol_1_No_2_December_2014/7.pdf

[38] Anderson JM, McCann EK (2002). Toward a post-colonial feminist methodology in nursing research: exploring the convergence of post-colonial and black feminist scholarship. Nurse Res.

[39] Racine L (2003). Implementing a postcolonial feminist perspective in nursing research related to non-western populations. Nurs Inq.

[40] O’Mahony J, Donnelly T, Raffin Bouchal S, Este D (2013). Cultural background and socioeconomic influence of immigrant and refugee women coping with postpartum depression. J Immigr Minor Health.

[41] Kramer EJ, Kwong K, Lee E, Chung H (2002). Cultural factors influencing the mental health of Asian Americans. West J Med.

[42] Saraceno B, van Ommeren M, Batniji R, Cohen A, Gureje O, Mahoney J (2007). Barriers to improvement of mental health services in low-income and middle-income countries. Lancet.

[43] World Health Organisation, Department of Health (2012). Health service delivery profile: Philippines [internet].

[44] Education First. EF English Proficiency Index [Internet]. Web only: Education First Ltd; 2015 [cited 2016 May 11]. Report No.: 5th Edition. Available from: http://mediakey1.ef.com/__/~/media/centralefcom/epi/downloads/full-reports/v5/ef-epi-2015-english.pdf.

[45] Semics LLC (2007). Culture and health among Filipinos and Filipino-Americans in Central Los Angeles [internet].

[46] Carlsen B, Aakvik A (2006). Patient involvement in clinical decision making: the effect of GP attitude on patient satisfaction. Health Expect.

[47] Sanchez F, Gaw A (2007). Mental health care of Filipino Americans. Psychiatr Serv.

[48] Al-Natour RJ. The Impact of the Researcher on the Researched. MC J [Internet]. 2011 [cited 2017 Sep 5];14(6). Available from: http://www.journal.media-culture.org.au/index.php/mcjournal/article/view/428.

[49] Norwegian Ministry of Health and Care Services (2013). Equitable health care service: good health for everyone [Norwegian] [internet].

[50] Donnelly TT, McKellin W. Keeping healthy! Whose responsibility is it anyway? Vietnamese Canadian women and their healthcare providers’ perspectives. Nursing inquiry. 2007;14(1):2–12. 10.1111/j.1440-1800.2007.00347.x 17298603

[51] David E, Nadal KL. The colonial context of Filipino American immigrants’ psychological experiences. Cultur Divers Ethnic Minor Psychol. 2013;19(3):298–309. 10.1037/a0032903 23875854

[52] Weerasinghe S, Mitchell T (2007). Connection between the meaning of health and interaction with health professionals: caring for immigrant women. Health Care Women Int.

[53] Corrigan PW, Morris SB, Michaels PJ, Rafacz JD, Rüsch N (2012). Challenging the public stigma of mental illness: a meta-analysis of outcome studies. Psychiatr Serv.

[54] Rüsch N, Angermeyer MC, Corrigan PW (2005). Mental illness stigma: concepts, consequences, and initiatives to reduce stigma. Eur Psychiatry.

