# Supplementary material for: “It has not occurred to me to see a doctor for that kind of feeling”: a qualitative study of Filipina immigrants’ perceptions of help seeking for mental health problems
Source: BMC Womens Health. 2018 May 25;18:73. doi: 10.1186/s12905-018-0561-9 (PMC5970497; doi:10.1186/s12905-018-0561-9)
Supplement: Supplementary file 1 — Interview guide (English): List of questions and probes used during interviews. (DOCX 17 kb) [file 12905_2018_561_MOESM1_ESM.docx]

**Interview Schedule**

1. **Can you tell me about yourself and how you came to live in Norway?**

Probes: where were you born, when did you move, why? Have you lived in other countries?

Situation now - Marriage / family / children – in Norway and at home. Responsibility for family at home?

Employment history – before and after moving.

Long term plans – staying in Norway?

1. **How do you feel about living in Norway?**

Probes: How well do you feel adjusted? Role of husband and other family members / friends / social network / work.

Good / bad aspects of living in Norway (e.g. being away from family, climate, food, language, work, people, social network, racism, responsibility for family etc). How have you overcome these problems? What helped?

1. **How do you manage to balance different responsibilities at work and at home and responsibilities for your family in your home country? (if applicable)**

Probes: Did you find it stressful? How did it affect you? How have you been able to resolve this?

1. **Have you ever felt sad or low while in Norway?**

Probes: can you give me an example of when? How did you feel, how did it affect you? For how long? What happened? Did it influence your daily life?

1. **What helped you get through it?**

Probes: Family/friends/work/understanding the culture/language/time? Hobbies or other activities? Did you talk to anyone about the way you felt? Friend, family, husband, professional help? Did you look for practical help? What changed about the situation?

1. **I am wondering a little about cultural health practices in your home country compared with Norway…**

Probes: What are the normal practices when someone is ill at home? E.g. cold/flu. How is it treated? When would you consult a doctor? What about more serious or chronic illnesses? How does someone stay healthy at home? Role of diet, exercise etc.

1. **What about perceptions of mental health?**

Probes: Why might one have a mental health problem? How would someone cope with their problems? What kind of help might someone get? What might prevent mental health problems? What does depression mean to you? How might someone with depression act?

Do you think it is different in Norway from home? In what ways? Coping strategies? Expressions of stress or depression – somatic problems – headaches, pain, stomach ulcers. Sleep, tiredness, appetite etc.

1. **(If indications of stress or feeling low) Do you think you have ever experienced a mental health problem (or that you could have had you continued in the same situation you were in)? Or have you ever had a diagnosed mental health problem?**

Probes: if so, was this while in Norway? Can you tell me a little more about it… when did it start, how did you feel, what symptoms did you have? (e.g. sleep, appetite, tiredness, headaches, pain etc.) How did it affect your daily life? How did you cope with it? What kind of help did you get if any? Did your family know about it?

1. **Have you ever had physical symptoms such as headaches, stomach aches, muscular pain that have not had a physical explanation?**

Probes: can you tell me more about it? Did you experience changes in sleep patterns, appetite, tiredness levels too? What might cause this?

1. **Moving on to talk a bit about your experiences with the health care system in Norway. How easy do you think it is to visit the doctor here?**

Probes: difficulties making an appointment, getting to the doctors, other barriers such as cost, time, travel, work etc. Waiting for appointment, prioritising problems. Have you used any health care other services in Norway? E.g. out of hours (legevakt), hospital, community health centre (helsestasjon)?

1. **How easy was it to understand how the health system in Norway works?**

Did you get help from anyone? E.g. spouse, friend, boss, colleague, Norwegian class? How did you get a GP? Have you had more than one GP since you’ve been here? Navigating the health system – gatekeeper role versus direct entry to secondary services. What might make it easier for immigrants?

1. **How do you find it actually being at the doctors during a consultation? Do you get the help you expect?**

Probes: How often do you visit the doctor? Does he/she listen to your problems? Are you pleased with the way the problems are resolved? Example – good and bad? Do you feel the doctor understands your needs? Do you ever experience communication problems? Example? Have you ever needed an interpreter when you’ve been at the doctors? Does this help your communication with the doctor? Do you think your doctor’s cultural background affects your communication?

1. **How do your experiences compare with visiting the doctor back at home?**

Probes: How do you arrange to see a doctor? How is it when you are at the doctors there? Does the doctor understand your needs? What are the main differences in visiting the doctor at home?

Have you visited the doctor or other health care services at home since moving to Norway?

1. **Have you or do you think you could visit your doctor here in Norway if you were having emotional problems, or were feeling stressed or depressed?**

Probes: Why/why not?

How do you think the doctor might be able to help?/What did the doctor do to help? Prescription medicine, sickness absence, conversational therapy, referrals to psychologist, telephone contacts.

Alternative: What about if a friend was experiencing problems? For instance, if they were depressed / very sad and low? What might you encourage them to do?

1. **Where else might you look for help if you were having emotional problems, or were feeling stressed or depressed?**

Probes: Would you discuss it with your spouse? Family or friends in Norway? Family/ friends at home? Others in social network – school/work e.g. manager? Professionals? Helpline? A religious leader or group? Other social groups? Alternative medicine? Take up hobbies /other activities? In what ways do you think these people might be able to help / offer support? Practical support to change the situation causing stress?

1. **Is there anything else you want to add about health care or mental health that you’ve not had chance to say yet?**
2. **Can I ask how you felt about being interviewed today?**
