# Supplementary material for: “It has not occurred to me to see a doctor for that kind of feeling”: a qualitative study of Filipina immigrants’ perceptions of help seeking for mental health problems
Source: BMC Womens Health. 2018 May 25;18:73. doi: 10.1186/s12905-018-0561-9 (PMC5970497; doi:10.1186/s12905-018-0561-9)
Supplement: Supplementary file 2 — Questionnaire (English): Questionnaire for collecting background information. (DOCX 20 kb) [file 12905_2018_561_MOESM2_ESM.docx]

Please complete the following information:

**Name:** ____________________

**Age** (years):

**Citizenship**: _____________

**Ethnicity**: ____________________

**How long have you**

**lived in Norway?** (years)

**Do you have any children?**

□ Yes □ No

**Were any of your children born in Norway?**

□ Yes □ No

**Employment status**:

□ Full time employment (30+ hrs. per week)

□ Part time employment (<30 hrs. per week)

□ Student

□ Homemaker

□ Unemployed/ Seeking work

□ Retired/Disability pension

□ Other: ______________

**Civil status**:

□ Married / cohabiting

□ Separated /divorced

□ Widowed

□ Single

**Highest completed level of education**:

□ Primary/ elementary school

□ Lower secondary/ middle school

□ Upper secondary / high school

□ College / University

□ Other:_________

**Listed below are some symptoms or problems that people sometimes have. Please read each one carefully and decide how much the symptoms bothered or distressed you during the last week, including today?** (Check the appropriate column)

|  | Not  at all | A little | Quite  a bit | Extremely |
| --- | --- | --- | --- | --- |
| Suddenly scared for no reason |  |  |  |  |
| Feeling fearful |  |  |  |  |
| Faintness, dizziness or weakness |  |  |  |  |
| Feeling tense or keyed up |  |  |  |  |
| Blaming yourself for things |  |  |  |  |
| Difficulties falling asleep or staying awake |  |  |  |  |
| Feeling blue |  |  |  |  |
| Feelings of worthlessness |  |  |  |  |
| Feeling everything is an effort |  |  |  |  |
| Feeling hopeless about the future |  |  |  |  |
