# Supplementary material for: “It has not occurred to me to see a doctor for that kind of feeling”: a qualitative study of Filipina immigrants’ perceptions of help seeking for mental health problems
Source: BMC Womens Health. 2018 May 25;18:73. doi: 10.1186/s12905-018-0561-9 (PMC5970497; doi:10.1186/s12905-018-0561-9)
Supplement: Supplementary file 3 — Questionnaire (Norwegian): Questionnaire for collecting background information. (DOCX 20 kb) [file 12905_2018_561_MOESM3_ESM.docx]

Vennligst fyll ut følgende informasjonen:

**Navn:** ____________________

**Alder** (år):

**Statsborgerskap**: _____________

**Etnisitet**: ____________________

**Hvor lenge har**

**du bodd i Norge** (år)?

**Har du barn?**

□ Ja □ Nei

**Har du barn som er født i Norge?**

□ Ja □ Nei

**Arbeidsstatus** (sett kryss):

□ Inntektsgivende arbeid (30+ timer i uka)

□ Deltidsarbeidende (<30 timer i uka)

□ Student

□ Hjemmearbeidende

□ Arbeidsløs/ søkende

□ Pensjonist/ufør

□ Annet:………………….

**Sivilstand** (sett kryss):

□ Gift / samboer

□ Separert /skilt

□ Enke

□ Singel

**Høyeste utdanning fullført**:

□ Grunnskole/Folkeskole

□ Framhaldsskole, realskole

□ Videregående skole

□ Høyskole / Universitet

□ Annet:……………..

**Under finner du en liste over ulike problemer. Har du opplevd noe av dette den siste uken (til og med i dag)?** *(Sett ett kryss for hver plage)*

|  | Ikke plaget | Litt plaget | Ganske mye | Veldig mye |
| --- | --- | --- | --- | --- |
| Plutselig frykt uten grunn |  |  |  |  |
| Føler deg redd eller engstelig |  |  |  |  |
| Matthet eller svimmelhet |  |  |  |  |
| Føler deg anspent eller oppjaget |  |  |  |  |
| Lett for å klandre deg selv |  |  |  |  |
| Søvnproblemer |  |  |  |  |
| Nedtrykt, tungsindig |  |  |  |  |
| Følelse av å være unyttig, lite verd |  |  |  |  |
| Følelse av at alt er et slit |  |  |  |  |
| Følelse av håpløshet mht. framtida |  |  |  |  |
